# Complexity of Multi-Dimensional Spontaneous EEG Decreases during Propofol Induced General Anaesthesia

**DOI:** 10.1371/journal.pone.0133532

**Published:** 2015-08-07

**Authors:** Michael Schartner, Anil Seth, Quentin Noirhomme, Melanie Boly, Marie-Aurelie Bruno, Steven Laureys, Adam Barrett

**Affiliations:** 1 Sackler Centre for Consciousness Science, Department of Informatics, University of Sussex, Brighton, United Kingdom; 2 Coma Science Group, University of Liège, Liège, Belgium; 3 Department of Neurology, University of Wisconsin, Madison, United States of America; 4 Department of Psychiatry, University of Wisconsin, Madison, United States of America; 5 Department of Clinical Sciences, University of Milan, Milan, Italy; National Scientific and Technical Research Council (CONICET)., ARGENTINA

## Abstract

Emerging neural theories of consciousness suggest a correlation between a specific type of neural dynamical complexity and the level of consciousness: When awake and aware, causal interactions between brain regions are both integrated (all regions are to a certain extent connected) and differentiated (there is inhomogeneity and variety in the interactions). In support of this, recent work by Casali et al (2013) has shown that Lempel-Ziv complexity correlates strongly with conscious level, when computed on the EEG response to transcranial magnetic stimulation. Here we investigated complexity of spontaneous high-density EEG data during propofol-induced general anaesthesia. We consider three distinct measures: (i) Lempel-Ziv complexity, which is derived from how compressible the data are; (ii) amplitude coalition entropy, which measures the variability in the constitution of the set of active channels; and (iii) the novel synchrony coalition entropy (SCE), which measures the variability in the constitution of the set of synchronous channels. After some simulations on Kuramoto oscillator models which demonstrate that these measures capture distinct ‘flavours’ of complexity, we show that there is a robustly measurable decrease in the complexity of spontaneous EEG during general anaesthesia.

## Introduction

The idea that the level and range of consciousness relates in some way to dynamical complexity of brain activity is becoming increasingly prominent [1–7]. A common way to conceptualize dynamical complexity in this setting is as simultaneous *differentiation* (subsets of the system being dynamically distinct) and *integration* (the system as a whole exhibiting coherence), and this idea draws from what is taken to be a fundamental property of conscious experience, namely that each conscious scene is composed of many different parts and is different from every other conscious scene (differentiation), yet each conscious scene is experienced as a coherent whole (integration) [2–5]. A number of different measures of neural dynamical complexity have been proposed based on information sharing and transfer [2, 4, 8–14]. Properties of these measures have been explored on simple models, for instance neural network activity of artificial agents [9, 15]. However, these measures, based on information sharing and transfer, rely on restrictive assumptions, such as stationarity and linearity, that limit the conclusions that can be drawn when applied to real brain data [16, 17].

A series of recent studies take a more pragmatic approach to investigating the relationship between consciousness and complexity [18–22]. These studies have investigated, for subjects in diverse states of consciousness (e.g. wakeful rest, deep sleep, general anaesthesia), the electroencephalographic (EEG) response to transcranial magnetic stimulation (TMS). It has been found that, when subjects are unconscious, the response is stereotypical across electrodes and remains local to the site of stimulation, whereas when subjects are conscious, the response differs between electrodes and spreads across the whole cortex. In this way, the extent of both differentiation and integration is greater when the subject is conscious. A simple measure of complexity based on the (lack of) compressibility of the EEG response, as quantified by the Lempel-Ziv algorithm [23], was developed for these data: the Perturbational Complexity Index (PCI) [21]. The PCI reflects simultaneous integration and differentiation since the EEG response is least compressible when it is both widespread and inhomogeneous. In a first application of this method, the PCI values obtained for conscious subjects were consistently higher than for unconscious subjects, to the extent that a single classifier threshold could be applied: when the PCI value was above the threshold the subject was always conscious and when the PCI value was below the threshold the subject was always unconscious [21].

On spontaneous steady-state EEG data, several indices labelled as measures of complexity have been computed on single time-series, reflecting local signal diversity over time rather than differentiation and/or integration across a network. These measures include various forms of spectral entropy [24–26] and again Lempel-Ziv complexity [27–35] and all of them have a tendency to decrease during general anaesthesia. Recently these measures have also been applied to auditory evoked potentials in disorders of consciousness patients [36], and values were found to correlate with behaviourally-diagnosed level of consciousness, although there was no single cross-subject threshold for classifying subjects as conscious or unconscious.

Here we investigated three simple measures of complexity on multi-dimensional spontaneous steady-state EEG data from subjects undergoing propofol-induced general anaesthesia. The first of these is the aforementioned Lempel-Ziv complexity. On spontaneous data Lempel-Ziv complexity strictly only reflects differentiation (and not integration); it computes diversity in patterns of activity in both space and time. The other two measures are amplitude and synchrony coalition entropy (respectively ACE and SCE). ACE is a variant of a measure introduced by Shanahan in [37], and reflects the entropy over time of the constitution of the set of most active channels, while the novel SCE reflects the entropy over time of the constitution of the set of synchronous channels. ACE is similar to Lempel-Ziv complexity, in the sense that it quantifies variability in space and time of the activity. By contrast, SCE is conceptually different because it quantifies variability in the relationships between pairs of channels.

We computed Lempel-Ziv complexity and the coalition entropy measures for sets of equally spaced channels across the whole scalp and also for sets of channels restricted respectively to the frontal, parietal, temporal and occipital lobes, on full broadband signals and on frequency-restricted signals, in each case comparing results for data from wakeful rest, mild sedation and general anaesthesia. We contrasted these measures’ ability to indicate conscious level on these data, with that of control measures not based on signal complexity, including normalized delta power [36, 38, 39]. In order to facilitate interpretation of our results we also computed the measures on simulated data from (i) random matrices with varying numbers of identical signals; (ii) a modular network of Kuramoto oscillators, as in [37].

## Methods

### Ethics statement

The data analysed in this study were obtained from a previous study [38] with procedures approved by the Ethics Committee of the Faculty of Medicine of the University of Liège.

### EEG data acquisition and preprocessing

Spontaneous high-density EEG recordings (256 electrodes, EGI [40]) were re-analysed from 7 healthy subjects, sampled at 1000*Hz*, before, during and after propofol-induced general anaesthesia. Propofol is an intravenous anaesthetic that is widely used in surgical settings and which reversibly induces a state of diminished responsiveness behaviourally similar to non-rapid eye movement sleep [41]. States of consciousness were defined behaviourally using the Ramsay scale [42]. The 4 different states labelled here are: wakeful rest (WR), mild sedation (MS; slower response to command, Ramsay scale score 3), loss of consciousness (LOC) with clinical unconsciousness (no response to command, Ramsay scale score 5), and recovery of consciousness (wakeful rest after propofol, WRa) [38]. Propofol was administered as described in [38]: ‘A simple constant rate infusion of propofol was used together with the computerized Marsh model to predict when to manually adjust infusion rate to maintain predicted steady-state propofol levels, although the main goal was to achieve the range of clinical states (Ramsey scores).’ Average arterial blood concentrations of propofol were 1.91±0.52*mcg*/*mL* for MS and 3.87±1.39*mcg*/*mL* for LOC [38]. 20 minutes of recording during each of these 4 states were obtained for each subject.

The data were pre-processed as follows. First epochs of recording and complete channels that showed obvious artefacts were rejected by visual inspection. Artefacted channels were identified by their extreme amplitudes and irregular behaviour throughout the complete recording and entirely removed. Artefacted epochs, displaying abnormally high amplitudes for several seconds across all channels (typical for muscle movement), were manually excised and the remaining data concatenated. Then 50*Hz* and 100*Hz* frequency noise (artefacts from electricity mains) were removed by Butterworth notch filtering. Next the data were down-sampled from 1000*Hz* to 250*Hz* and spatially filtered by computing the surface Laplacian.

Surface Laplacian is a method for spatial filtering of EEG data, performed in order to increase topographical specificity (i.e. to make each channel’s activity more directly indicative of brain activity under the channel’s electrode). It reduces the effect of volume-conduction (electrical fields tangentially conducted along the skull) so the filtered signal reflects more closely the local brain activity—radial dipoles in gyral crowns [43]. For each electrode, the surface Laplacian can be implemented as a subtraction of a weighted sum of its nearest neighbours’ electrical activity. This filtering method was applied using MATLAB with BCIlab and EEGlab [44].

**Fig 1:**
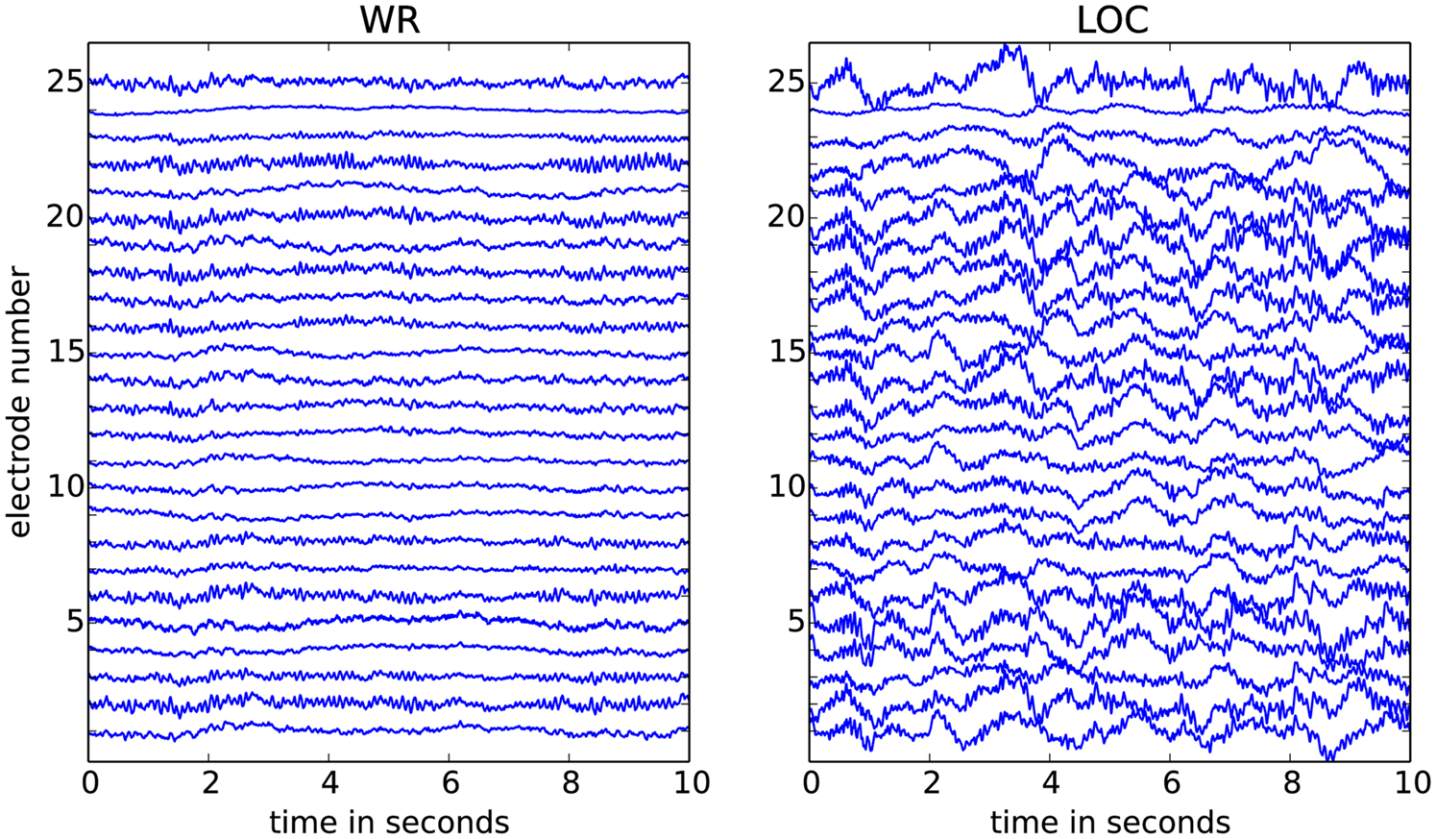
Two 10sec EEG segments from 25 channels. The segment in the left panel is during wakeful rest (WR) and the segment in the right panel is during propofol-induced loss of consciousness (LOC); both segments are shown after pre-processing. The voltage scale is the same for both conditions and the maximal fluctuation shown is approximately 0.1*mV* (for more data details, see [16, 38]). The recordings for LOC display visibly stronger slow waves (low-frequency components) as compared to those for WR.

Finally linear de-trending and baseline subtraction was performed for each channel of each segment. After preprocessing the length of the time series varied per subject and per condition between 9–14min, i.e. approximately half the length of the raw data. Fig 1 illustrates example 10sec segments of EEG data for WR and for LOC. Analyses were performed using such non-overlapping 10*sec* segments for a total number of on average 60 segments of EEG recording per subject and per condition.

### EEG channel selection

**Fig 2:**
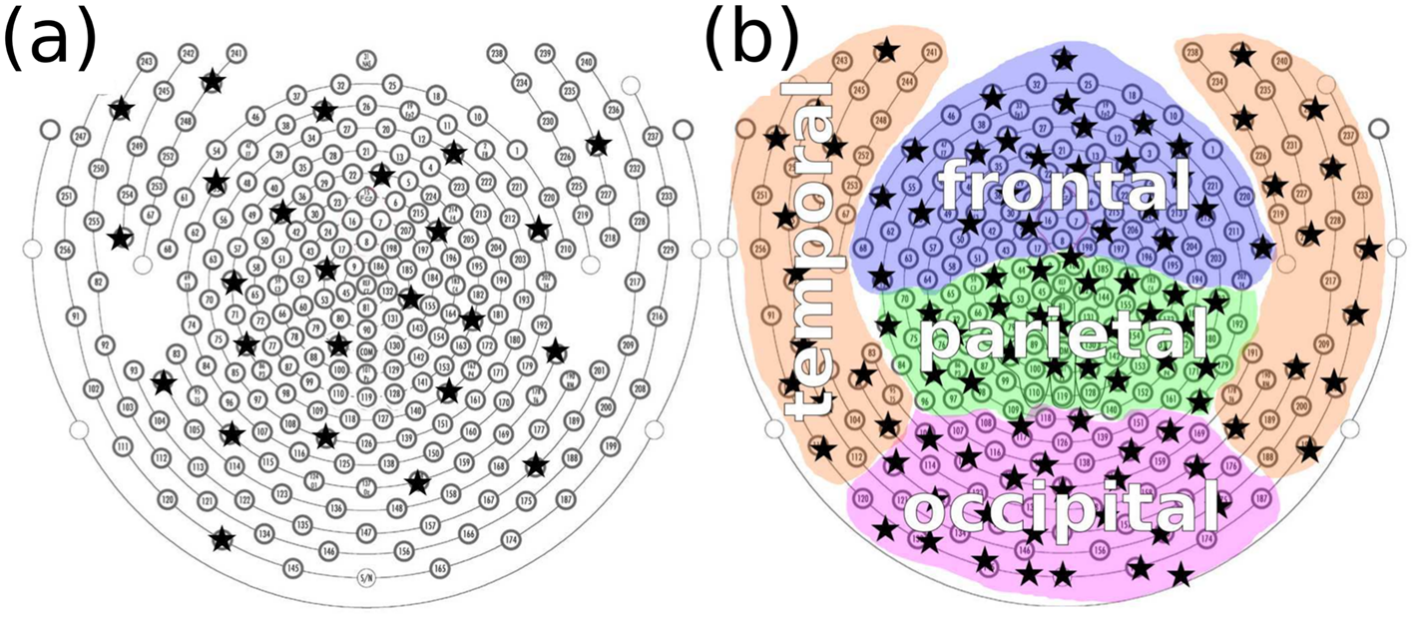
Channel selection from the high density (256 electrodes) EEG. The electrode layout is from manufacturer EGI [40]. The homogeneously distributed channel selection is shown for: a) analyses across the whole scalp; b) analyses restricted to a certain lobe. Chosen channels are indicated as black stars. See text for details.

Analyses were performed on automatically selected electrodes, first 25 taken from across the whole cortex, see Fig 2a, and secondly 25 taken exclusively from either frontal, parietal, temporal or occipital cortex, Fig 2b. A k-medoids clustering algorithm [45] was implemented in Python to automatically select electrodes spatially uniformly distributed over a particular region (either the whole cortex or a specific lobe).

The k-medoids cluster algorithm takes two arguments as input, first a set of 3D coordinates of points in space (i.e., the EEG electrode coordinates) and second the number of desired clusters. It outputs labels for each point, indicating to which cluster it belongs and also if it is this cluster’s representative. The cluster representative is the point in a cluster which has the least mean Euclidean distance to all other points of that cluster. The algorithm finds these labels by initialising cluster representatives as randomly chosen points of the set, as many as there are desired clusters. Then it iteratively adjusts labels until all points are divided into approximately equally large regional clusters. The representatives of the clusters are then approximately homogeneously distributed across the cloud of all points in space, irrespective of the cloud’s shape.

### Statistics

For within subject comparison of states, we considered differences in the scores of a measure to be substantial if the effect size as measured by Cohen’s *d* was greater than 0.8 (a threshold indicating large effect size [46]). For a given subject, measure and state pair, we computed Cohen’s *d* as the difference of the mean scores across segments for each state respectively, divided by the pooled standard deviation.

In addition we compared for each measure and state pair the mean scores across subjects. Given the independence of different subjects, we applied a Wilcoxon rank sum test for each measure and state pair, corrected for false discovery rate (FDR), using the Benjamini-Hochberg procedure.

### Lempel-Ziv complexity

**Fig 3:**
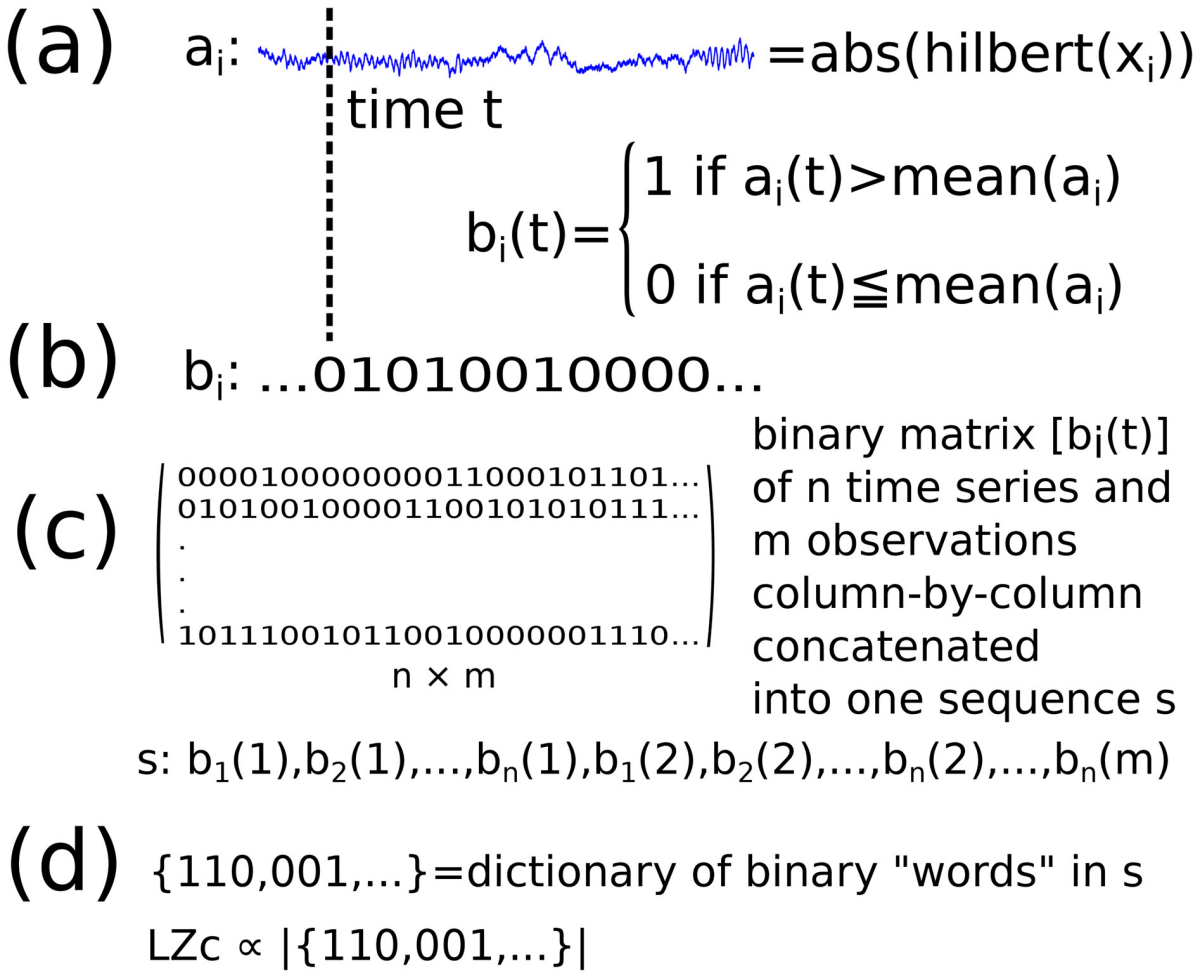
Schematic of the computation of Lempel-Ziv complexity LZc. a) *x*_*i*_ is the activity of the *i* ^*th*^ EEG electrode, i.e. the *i* ^*th*^ channel, and *a*_*i*_ is the (Hilbert) amplitude of *x*_*i*_. b) *b*_*i*_ is binarized *a*_*i*_, using the mean activity of *a*_*i*_ as binarisation threshold. c) After binarisation of all *n* channels, successive *n* × 1 dimensional observations are concatenated to obtain one binary sequence *s* in which patterns are searched and listed into a dictionary of binary words via a Lempel-Ziv algorithm. d) Lempel-Ziv complexity LZc is proportional to the size of this dictionary.

For a given segment of data, Lempel-Ziv complexity quantifies complexity by counting the number of distinct patterns of activity in the data. It can be thought of as being proportional to the size of a computer file containing the data, after applying a compression algorithm. Computing the Lempel-Ziv compressibility of data requires a binarization of the multidimensional time series. Casali et al [21] computed this measure on event-related, as opposed to resting data, and so used a threshold relative to pre-stimulus activity (baseline) to define the binarization. Here our threshold was based on the instantaneous amplitude of the Hilbert transform, i.e. the absolute value of the analytic signal of the channel’s time series. The threshold *T*_*i*_ for the *i* ^*th*^ channel was chosen as the mean of the absolute value of the analytic signal of the *i* ^*th*^ channel. The data segment is then treated as a binary matrix, with rows corresponding to channels (time series) and columns corresponding to time (observations). A Lempel-Ziv compression algorithm obtains a list of words (binary subsequences that appear at least once) in the data matrix, as sketched in Fig 3. The Lempel-Ziv complexity is then proportional to the number of binary words. The greater the degree of randomness, the greater the number of different sub-sequences that will be present, and thus the higher the Lempel-Ziv complexity.

Different implementations of Lempel-Ziv data compression were tested, all resulting in nearly identical outcomes (see section ‘LZ measures’ in S1 Document for variants), so here we present results from the fastest and simplest implementation, denoted LZc, (rather than precisely the one used by Casali et al [21]). LZc is obtained by rearrangement of the binarized multidimensional time series, observation by observation, into a binary sequence as described in (c) in Fig 3 and then applying a standard open source Lempel-Ziv compression algorithm [47] to this sequence. LZc tells us how much variety there is in the patterns of activations.

We normalize LZc by dividing the raw value by the value obtained for the same binary input sequence (*s* in (c) of Fig 3) randomly shuffled. LZc’s raw score for a binary sequence of fixed length is maximal if the sequence is entirely random. (The variation in LZc for 50 different shufflings of the same input sequence was under 0.002% of the mean result across those 50, showing that the data matrices we analysed were sufficiently large such that there was negligible variance arising from basing the normalization on just a single random shuffling.) Thus the normalized LZc values indicate the level of complexity on a scale of 0 to 1. Note that Casali et al [21] instead used the asymptotic analytical upper bound as the normalization. This differs from the normalization used here because we require a maximal score of 1 for the same input randomized in time, which is the upper bound of the measure for the given input dimensions. See Section ‘LZ measures’ in S1 Document for details on the computation of LZc and comparison with PCI.

### Coalition entropy measures

The idea of coalition entropy was introduced by Shanahan [37, 48]. In its original form it measures the entropy (over time) of the constitution of the set (coalition) of channels that are active, given a binarization scheme for classifying channels as either active or inactive. We call this measure amplitude coalition entropy (ACE). Here we compute it using the same binarization as described for LZc, i.e. taking for each channel the mean of the absolute value of the analytic signal as the threshold. As for LZc we normalize ACE by dividing the raw value by the value obtained for the same binary input shuffled (the upper bound), where shuffling means that the position of each digit was randomly changed. Note Shanahan’s original version differs slightly from the version used here by utilizing a fixed absolute binarisation threshold (which is not applicable to real EEG data), and taking the asymptotic analytical upper bound as the normalization (which is again not reached for the shape of data matrix analyzed here, since the time-series are not long enough for all possible coalitions to be sampled). The entropy (over time) of the constitution of the set of channels that are active (assuming *n* channels in total) was implemented by mapping each of the possible *n* × 1 dimensional binary observations to a distinct integer and then computing the entropy of the resulting sequence of integers.

**Fig 4:**
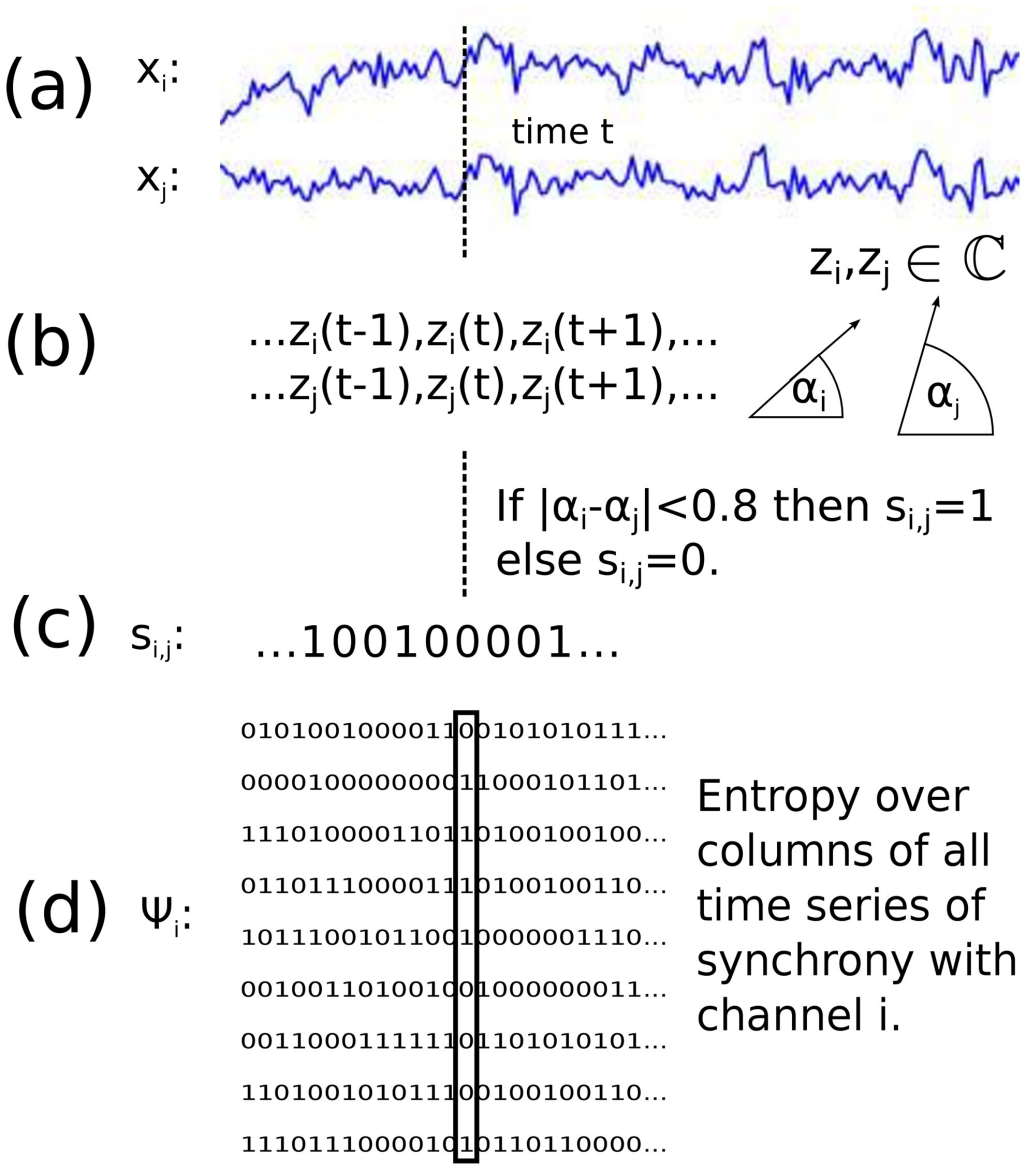
Schematic of the computation of SCE. a) Two time series. b) The analytic signals of these two, being complex signals with the real part being the original signal and the imaginary part being the Hilbert transform of the signal. c) A synchrony time series is created for this pair of signals, being 1 at time *t* if the phases of the complex values of the analytic signals have similar magnitude at this time *t*. d) SCE^(*i*)^ with respect to channel *i* is the entropy over columns (*n* × 1 synchronies) of the matrix Ψ_*i*_ containing all *n* synchrony time series for channel *i*. The overall SCE is then the mean value of the SCE^(*i*)^ across channels.

We also introduce a new variant of coalition entropy, synchrony coalition entropy, denoted SCE. This measures the uncertainty, and hence diversity, over time of the constitution of the set of channels that are in synchrony—rather than active—schematically described in Fig 4 and formally defined as follows:

For data **X**_*t*_, consisting of channels *X*_*i*,*t*_, *i* = 1, …, *n*, we consider two channels to be in synchrony at time *t* if the absolute value of the difference between their instantaneous Hilbert phases is less than 0.8 radians (approximately 45 degrees). Then we define coalition time-series ${\text{Ψ}}_{t}^{\left(i\right)}$ by $\Psi_{j,t}^{\left(i\right)}$ taking the value 1 if channels *i* and *j* are synchronised at time *t* and taking the value 0 otherwise. The coalition entropy of **X**_*t*_ with respect to channel *i* is the entropy of ${\text{Ψ}}_{t}^{\left(i\right)}$ (over time), normalized as a proportion of its maximum possible value *N*:

$$\begin{array}{c} {\text{SCE}}^{\left(i\right)}=-\frac{1}{N}\sum_{\psi }\;p\left(\Psi_{t}^{\left(i\right)}=\psi \right)\log\;p\left(\Psi_{t}^{\left(i\right)}=\psi \right)\;. \end{array}$$ (1)

The overall SCE is then the mean value of the SCE^(*i*)^ across channels. The upper bound SCE would arise from completely random coalition time-series in which each entry is 1 with probability 0.5. Such time-series are generated (with the same dimensions as those arising from the data) to obtain the normalization factor *N*. Note that SCE does not score exactly 1 for shuffled input data—unlike ACE and LZc—as the probability at a give time-point of two shuffled channels being in synchrony is less than 0.5.

Python implementation of all three complexity measures can be found in the Supplementary Information (S1 Code).

### Kuramoto model

In order to explore simulated time series for which SCE and ACE vary differently, we used a Kuramoto model. We used the same parameters as those in Shanahan [37] (with a small exception, see below). That is, we simulated 8 communities of 32 oscillators, with the activity of each oscillator being a unit complex number with phase *θ*. For the *i* ^*th*^ oscillator the evolution of its phase in time is given by:

$$\begin{array}{c} \frac{d\theta_{i}}{dt}=1+\frac{1}{N+1}\sum_{j=1}^{N}K_{i,j}\sin\left(\theta_{j}-\theta_{i}-\alpha_{i,j}\right). \end{array}$$ (2)

The matrix *K* of coupling strengths was such that each oscillator is fully connected to its own community and has 32 random connections to oscillators in other communities. *N* denotes the number of connections per oscillator. Inter-community coupling strengths were set to 0.4, and intra-community coupling strengths to 0.6. As shown in the equation, all natural frequencies are 1. An Euler method step size of 0.05 was used and 1500 time steps were generated, starting from random initial phases. The first 500 time steps were discarded. The phase lag parameter $\beta_{i,j}=\frac{\pi }{2}-\alpha_{i,j}$ was varied uniformly for inter-community connections, and held constant at 0.15 for intra-community connections. (Keeping the intra phase lag fixed is the only difference from the parameters in [37]). A small value of *β* corresponds to a large phase lag and leads to little synchrony whereas larger values of *β* correspond to smaller phase lags and leads to greater inter-community synchrony. A single time-series was generated for each community by taking the real part of the average instantaneous activity over all of its oscillators. Thus the activity *X*_*c*_(*t*) of the *c* ^*th*^ community *C*_*c*_ is given by

$$\begin{array}{c} X_{c}\left(t\right)=\text{Re}(\frac{1}{32}\sum_{j\in C_{c}}e^{i\theta_{j}\left(t\right)}). \end{array}$$ (3)

## Results

### Measure comparison on simulated time series

In the following we describe results from analyses of 2 differently generated time series. Firstly, analyses based on random time series constructed *a priori*, and secondly data generated by the Kuramoto model. These two different analysis show respectively: (i) each measure captures the degree of randomness in binary matrices of activations (LZc, ACE) or synchronies (SCE); (ii) there exists a scenario in which SCE diverges from LZc and ACE and thus captures a distinct ‘flavour’ of complexity.

**Fig 5:**
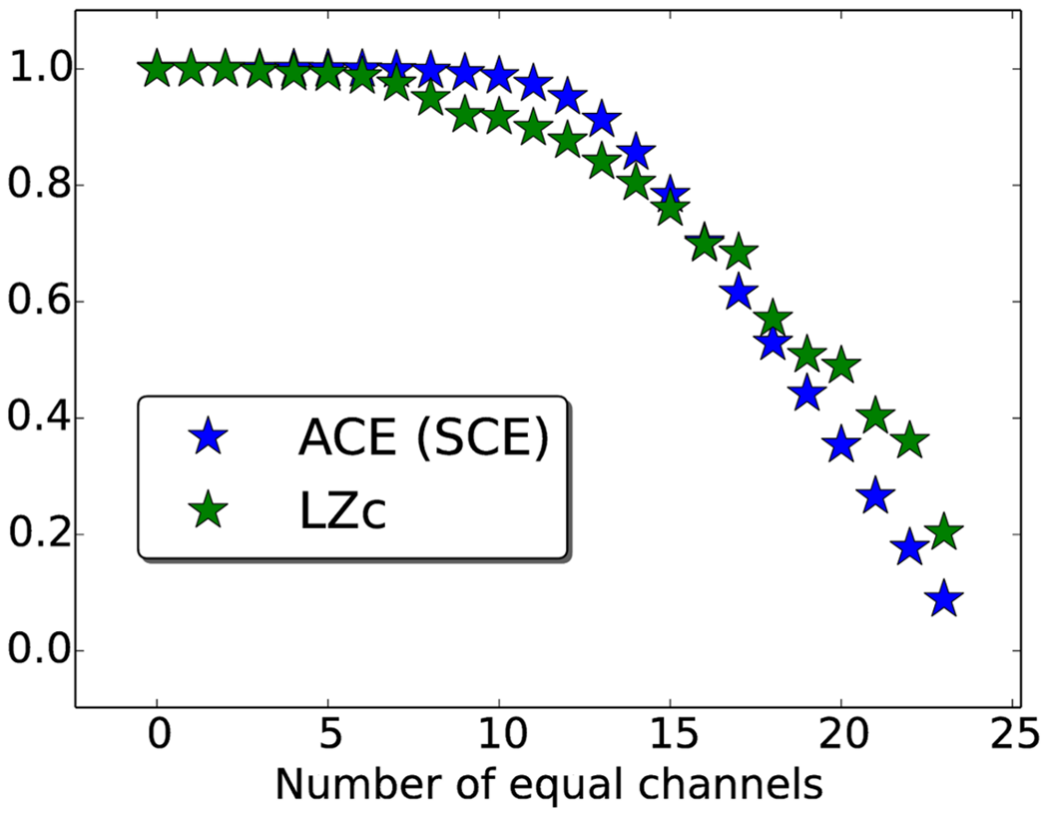
Complexity measures for increasingly regular activation/synchrony matrices. Given a random binary matrix of activations (for ACE and LZc) or synchronies (for SCE), with increasing number of duplicated channels, the complexity measures monotonically decrease with the number of equal channels. See text for details.

In the first simulation, we consider binary matrices, which represent the coalitions of either active (for LZc and ACE) or synchronous (for SCE) channels at each observation. We plotted the dependence of the measures against increasing order in this input matrix as follows. First a random binary matrix of 25 channels × 2500 observations was created (an entry being 1 with probability 0.5) and one by one each channel was replaced by a copy of the first channel. Thus at first the input matrix was fully random, then after one iteration there were two identical channels, at second iteration three identical channels (with random channel indices), until at the final iteration a matrix was obtained consisting of 25 identical channels. Coalition entropy and LZc all score 1 for the initial random matrix and decrease as the number of duplicate channels increases, approaching zero when all channels of the matrix are identical, see Fig 5 (repeated 100 times with identical results). Thus, as expected, LZc and ACE increase monotonically as the level of randomness of the matrix of activations increases, and so does SCE with the matrix of synchronies.

In the method just described, each channel remains fully random while the cross-channel variability is reduced. An alternative manipulation of randomness is to alter the regularity of each channel independently. We sorted increasingly long subsequences of each binary time series. That is, for each time series a subsequence of fixed length was chosen, starting at a different randomly chosen time in each time series. Then this subsequence of a given series is replaced by the same subsequence but ordered, placing all zeros to the left and all ones to the right within this subsequence. Now the length of the subsequence is increased stepwise, resulting in increasingly ordered binary matrices for which we computed the measures. We found the same trend as seen in Fig 5, with both LZc and coalition entropy (ACE and SCE) increasing as the level of randomness increases.

**Fig 6:**
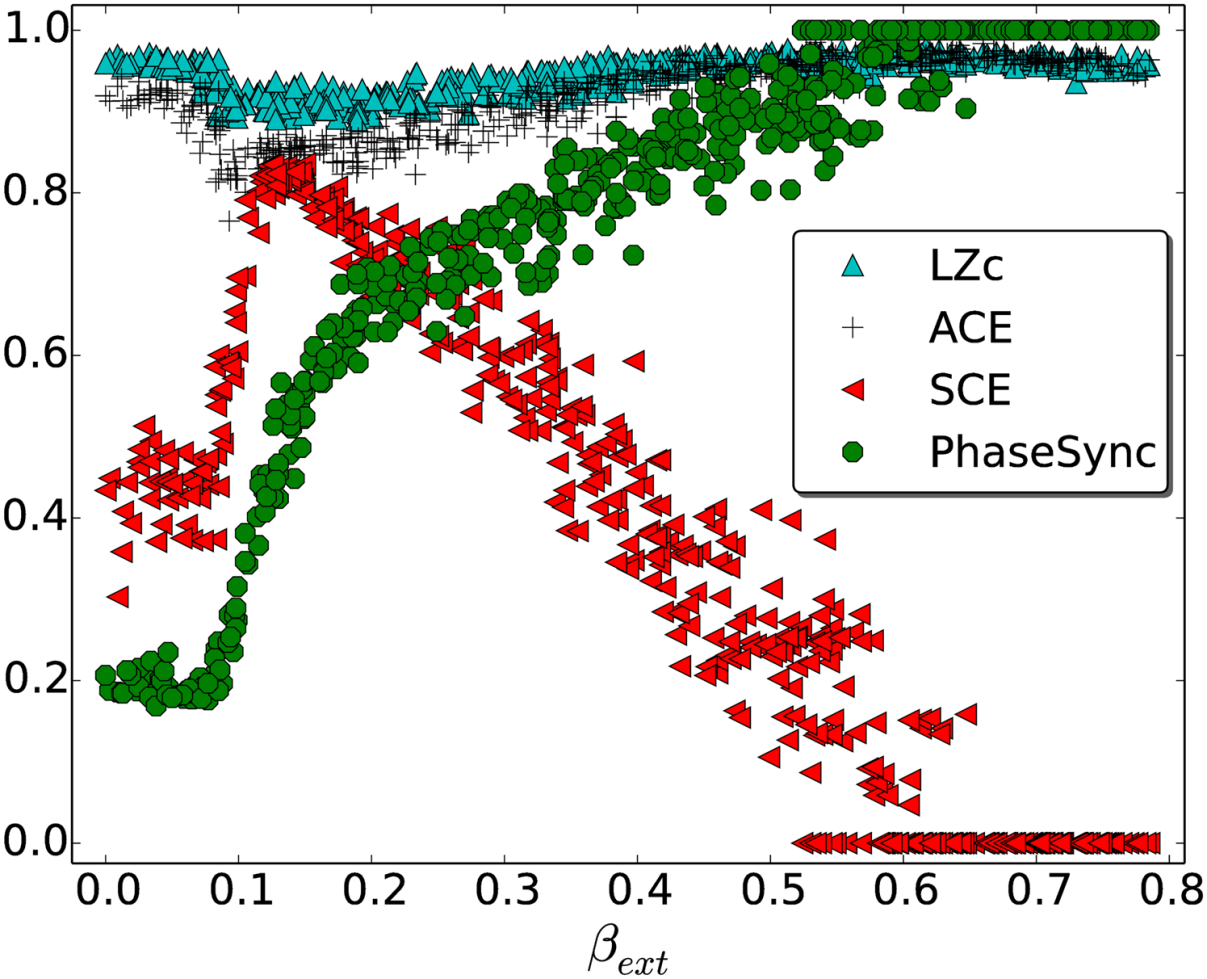
LZc, ACE and SCE for Kuramoto model. The data were obtained by varying phase-lags for inter-community interactions. A small value of *β*_ext_ corresponds to a large phase lag and leads to little inter-community synchrony (as measured by phase synchrony, PhaseSync—indicated as green discs, see text for its computation) whereas larger values of *β*_ext_ correspond to smaller phase lags and lead to greater inter-community synchrony. See main text for details. LZc and ACE show similar dependence on *β*_ext_ whereas SCE peaks where the former two have a minimum.

Secondly, we computed the three measures for simulated continuous data created with a Kuramoto model (see Methods, Section ‘Kuramoto model’), a model known to display rich dynamics [37, 49]. Fig 6 shows the results for the Kuramoto simulations, plotting LZc, ACE and SCE against the phase-lag parameter *β*_ext_. In addition a measure of phase synchrony is plotted (PhaseSync), being the mean of $\Psi_{t}^{\left(i\right)}$ over all observations *t* and channels *i*, as described in the definition of SCE. This plot illustrates (i) the similarity of LZc and ACE, which are both based on the diversity of the dynamics of amplitude fluctuations, and (ii) that SCE is a distinct measure of complexity to LZc and ACE, by virtue of being based on diversity in synchrony patterns. For all values of *β*_ext_, fluctuations in the amplitude of each community propagate to different communities, and this leads to diversity in relative amplitude reflected in the high values of LZc and ACE. In contrast, SCE is (i) low for small values of *β*_ext_ (large phase lag) since there is little synchrony between communities, (ii) low for large values of *β*_ext_ (small phase lag) since there is almost total synchrony between communities, (iii) high for intermediate values of *β*_ext_ in which synchrony between communities is able to fluctuate. Also note that LZc and ACE have a minimum at *β*_ext_ = 0.15 whereas SCE has a maximum there.

### Broadband signal for whole cortex

**Fig 7:**
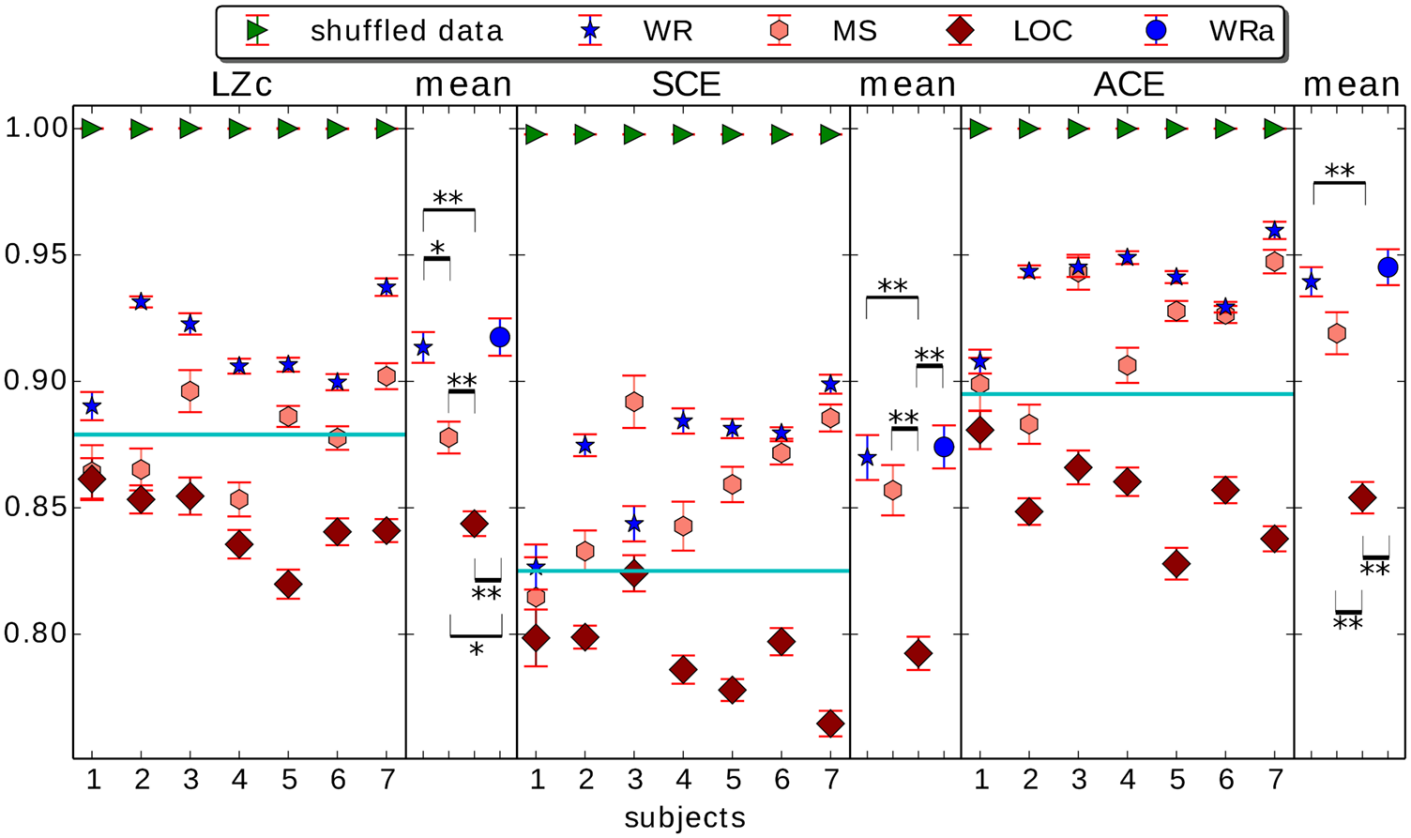
LZc, SCE and ACE computed as averages over multiple 10sec segments of EEG of the 7 subjects before and during anaesthesia. States shown are wakeful rest (WR) before propofol, mild sedation (MS), LOC and wakeful rest emerging from propofol sedation (WRa, not shown for single subject results). Measures are computed across 25 channels spread evenly across the whole cortex. The measures score highest for shuffled WR data, and consistently across subjects higher for WR as opposed to LOC. Error bars indicate standard error across segments, cyan horizontal lines are example thresholds for each of the measures, separating WR from LOC for all 7 subjects. For each single subject plot, the mean and standard error across its 7 values per state is displayed in the narrow plot to its right, with the title ‘mean’. For these mean values across subjects, significant differences between state pairs are shown by a double asterisk if *p* < 0.01 and a single asterisk if *p* < 0.05 (Wilcoxon rank sum test, FDR corrected for multiple comparison). See Table 1 for effect size comparison.

Fig 7 shows the mean values across 10*sec* segments of LZc, SCE and ACE during wakeful rest before sedation (WR), mild sedation (MS), LOC and shuffled data (in time domain shuffled WR) for each subject, computed for 25 EEG channels automatically selected to be spread evenly via k-medoids clustering across the whole cortex. For all subjects, the three measures LZc, SCE and ACE score higher for WR than for LOC, nearly all with high effect size (Cohen’s *d* > 0.8 for all measures and subjects except subject 1 for all measures and subject 3 for measure SCE, compare with Table 1.) Values for MS typically lie between those for WR or LOC, but the differences between MS vs. WR or LOC are less consistent than those between WR vs. LOC. For all three measures a single threshold can be drawn that separates WR from LOC across all subjects, as indicated by the cyan lines.

**Table 1:** Effect size comparison per measure and state pair.

	*γ*	*β*	*α*	*θ*	*δ*	sumCov	LZc	SCE	ACE
WR/WRa	1 4 2	0 1 6	4 3 0	6 1 0	2 4 1	0 7 0	0 6 1	1 6 0	0 6 1
WR/MS	4 2 1	0 0 7	6 1 0	7 0 0	2 2 3	0 3 4	4 3 0	1 6 0	2 5 0
WR/LOC	6 0 1	2 1 4	3 0 4	5 1 1	0 1 6	0 1 6	6 1 0	5 2 0	6 1 0
WRa/MS	5 2 0	0 2 5	3 3 1	5 2 0	1 3 3	0 5 2	2 5 0	1 6 0	2 5 0
WRa/LOC	6 0 1	4 1 2	1 1 5	4 1 2	0 1 6	0 2 5	6 1 0	6 1 0	6 1 0
MS/LOC	6 0 1	6 0 1	1 1 5	4 0 3	0 1 6	0 7 0	3 4 0	5 2 0	5 2 0

For each measure and state pair, the three numbers correspond to how many subjects out of 7 had higher score for the left state with Cohen’s *d* > 0.8 (left digit), no substantial difference, *d* < 0.8, (middle) and higher score for the right state with Cohen’s *d* > 0.8 (right). The results were obtained from applying the measures to the broadband signal from 25 k-medoids chosen electrodes from the whole cortex. Here WRa is wakeful rest emerging from propofol sedation.

Fig 7 further displays the mean scores of each measure—LZc, SCE and ACE—across the 7 subjects. By these mean scores, all three measures score higher for waking and mild sedation states (WR, WRa, Ms), than for LOC (*p* < 0.01, Wilcoxon rank sum test corrected for false-discovery rate using the Benjamini-Hochberg method). Also all three measures score higher for WR and WRa than for MS, though only significantly so for LZc (*p* < 0.05, see Fig 7).

The scores for all three states of consciousness and all three measures lie within 25% of the scores obtained for random input, see Fig 7. This proximity of the measures’ scores to 1 for WR (as well as LOC) contrasts with the pattern of the overall lower PCI scores presented by Casali et al for EEG input from propofol anaesthetized subjects (Fig 4a in [21]). There are however several differences between PCI and our measures, see, section ‘LZ measures’ S1 Document. Notably, PCI is computed after ordering the channels according to their overall level of activation following the TMS stimulation. This ordering reduces complexity. There is no analogous step in the computation of LZc, ACE and SCE. By design, all channels are equally ‘active’ according to the thresholding procedure we use on the spontaneous data.

We compared the ability of the complexity measures to discriminate the different states of consciousness with that of normalized spectral power bands and a simple correlation measure, sumCov, which equals the mean of the absolute values of correlation coefficients between all channels. Normalized spectral power bands were obtained by fast Fourier transform of each channel’s 10*sec* time series, then averaged over the 25 channels and grouped into frequency intervals, normalized such that the summed power across all bands equals one. Frequency bands are defined, following convention, as *δ* = 1–4Hz, *θ* = 4–8Hz, *α* = 8–13Hz, *β* = 13–30Hz, *γ* = 30–70Hz. All results are summarized in Table 1. The compared states were WR, MS, WRa (wakeful rest after propofol) and LOC.

We also assessed, via ROC curve analysis, the extent to which classification thresholds can be applied to each measure across subjects to discriminate between state pairs (e.g. WR versus LOC). Given the set of mean scores for each subject for a pair of states, one computes the ROC curve for discriminating between the states by plotting for each possible classification threshold the hit rate (*y*-axis) versus the false alarm rate (*x*-axis) when classifying a mean score as indicating the more conscious state when it exceeds the threshold. For example, in a comparison of LZc between WR and LOC, for a given threshold *C*, the hit rate is the proportion of subjects for which the mean LZc is greater than *C* during WR, and the false alarm rate is the proportion of subjects for which the mean LZc is greater than *C* during LOC. An area under the ROC curve (AROC) of 1 indicates that there exists a classification threshold for which for all 7 subjects the mean score exceeds the threshold for the more conscious state and is sub-threshold for the less conscious state. An AROC of 0 also indicates perfect discriminability between the states, but with the measure scoring higher for the less conscious state, i.e. there exists a threshold for which for all 7 subjects the mean score exceeds the threshold for the less conscious state and is sub-threshold for the more conscious state. An AROC of 0.5 indicates that the measure has no ability to discriminate between the states; for each threshold, the hit rate equals the false alarm rate. For an interactive applet that illustrates ROC analysis see [50].

**Fig 8:**
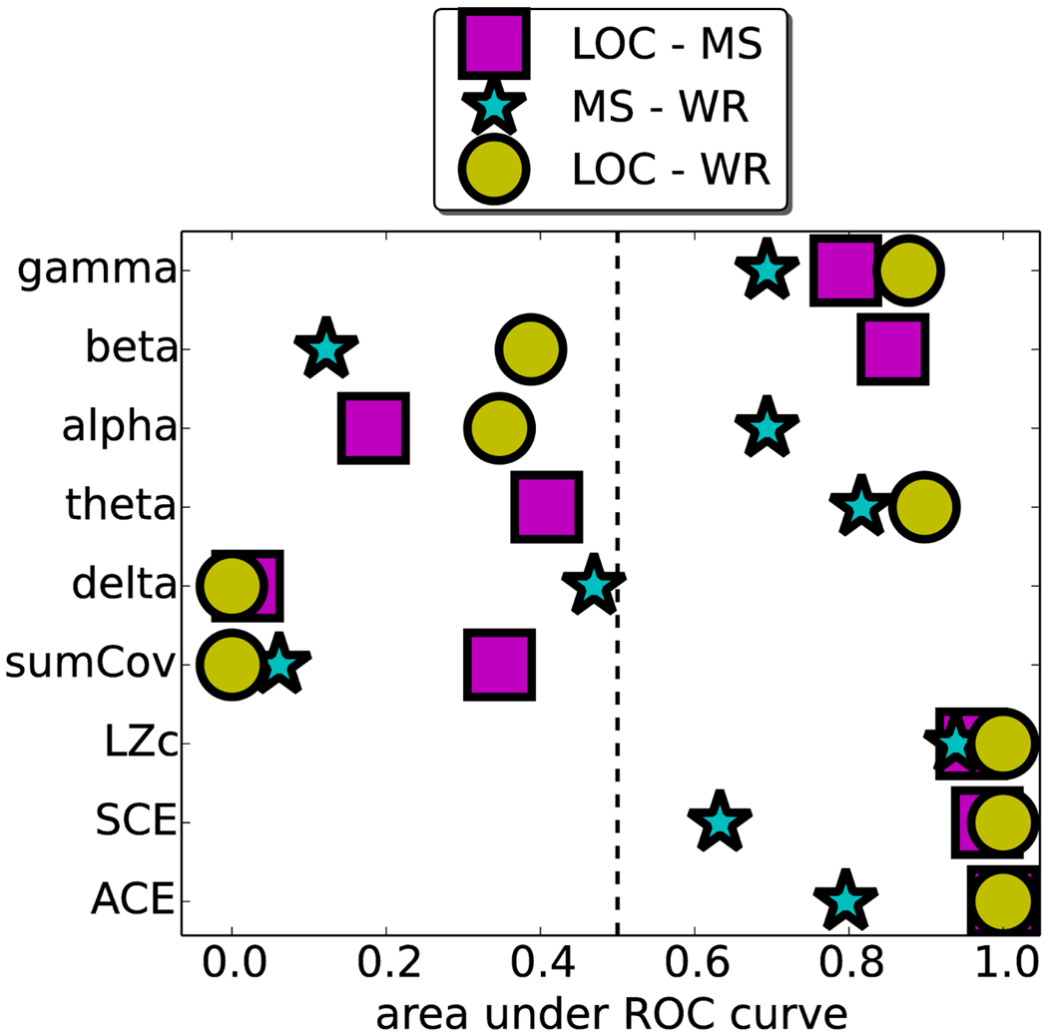
Discriminative power across subjects of nine measures, as measured by area under the ROC curve (AROC). Each symbol represents a state pair as indicated. The AROC is computed from the mean scores for the 7 subjects, obtained from the broadband signal from 25 electrodes from the whole cortex. The closer the AROC is to 0 or 1, the better the measure is at discriminating the given state-pair, close to 1 signifying that the measure tends to be greater for the more conscious state and close to 0 signifying that the measure tends to be greater for the less conscious state. When the AROC is 0.5 there is no discriminative power; hit rate equals false alarm rate for all classification thresholds. LZc, ACE and SCE have nearly maximal discriminative power for state pairs LOC/MS and LOC/WR. The measure sumCov fails to discriminate LOC/MS yet has strong (inverse) discriminative power for LOC/WR and MS/WR. Normalized delta band power discriminates LOC/WR and LOC/MS strongly yet MS/WR poorly.

As shown in Fig 8, LZc, SCE and ACE give nearly perfect cross-subject discrimination between LOC/WR and between LOC/MS, scoring lower for LOC than any other state. Discrimination was weaker for MS/WR. In addition, normalized delta power and sumCov give near perfect cross-subject discrimination between LOC/WR (delta also for LOC/MS, and sumCov also for MS/WR), scoring low for WR and highest for LOC. Delta band power strongly discriminating LOC/WR (see also section ‘Alternative measures’ in S1 Document and Table 1) is in line with previous studies [36, 38, 39], reflecting the presence of slow-waves during LOC [39].

#### Controlling for changes in power spectrum, effects of number of electrodes and segment length

Importantly, to check whether changes in ACE, LZc and SCE reflected more than mere changes to the power spectrum, we computed the measures normalised by their values for phase-randomised surrogate data. Phase shuffling was performed by Fourier transforming, and then applying inverse Fourier transform with the addition of an independent random phase to each channel. When repeating the analysis displayed in Fig 7 no major changes in the measures’ behaviour were found. (See section ‘Control for changes in power spectrum’ in S1 Document.)

Next, we tested dependence of the results on the number of electrodes across which the measures were computed. Different numbers of electrodes were chosen equally distributed across the whole cortex via k-medoids. We tested 5, 10, 50, and 100 electrodes in addition to the 25 considered above, for the measures LZc, SCE and ACE, all computed from 10*sec* segments. The results are all broadly the same as for 25 electrodes (see section ‘Dependence on channel number’ in S1 Document).

As a final control, the role of segment length was explored. Analysed across segments of length other than 10*sec*, we found for LZc, SCE and ACE broadly identical results in almost all cases for all tested segment lengths (0.2, 0.4, 0.8, 1.2, 2, 4, 6, 10 and 20 seconds). There was just one subject, subject 1, for whom LZc, SCE and ACE were only significantly greater in WR than LOC for segments of length 2*sec* or more. We conclude that the discriminative power of the measures is robust across a range of segment lengths. (See section ‘Dependence on segment length’ in S1 Document).

#### Correlation between changes in different measures

**Fig 9:**
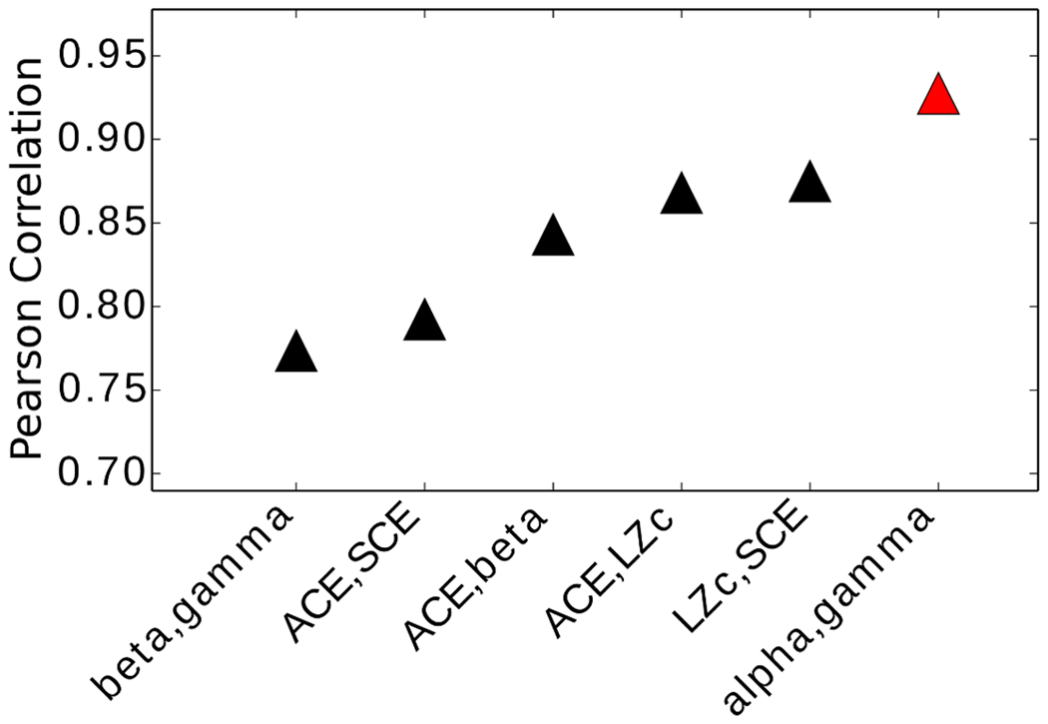
Pearson correlations between measures. Each black triangle indicates the positive correlation for the given measure pair listed in increasing order along the x-axis. The correlation was computed across subjects for the ratio of WR/LOC. Each correlation of all possible pairs of 9 measures was tested for significance and only those with 2-tailed *p*-value smaller than 0.05 are plotted. The red triangle indicates negative correlation. All 3 pairs out of ACE, LZc, SCE correlate significantly. There is no significant correlation of delta power with any other of the 9 measures for the state pair WR/LOC.

We computed correlations between all pairs of measures. The ratio of the score for WR and LOC was obtained for each subject and measure and then used to compute the Pearson correlation coefficient across subjects, as well as a 2-tailed *p*-value for that correlation. In other words, for each pair of measures we considered the correlation of measure1(LOC)/measure1(WR) and measure2(LOC)/measure2(WR) across subjects. For the state pair WR/LOC all correlations with *p* > 0.05 were ignored and the remainder are plotted in Fig 9, ordered by magnitude of the correlation. All pairs chosen from the measures ACE, LZc, SCE correlated significantly, with Pearson correlation coefficient (*r*) greater than 0.75 and *p* < 0.05. Beta power changes correlated strongly with changes to ACE, even though beta sometimes increases and sometimes decreases during LOC (see Table 1). All significant correlations were positive except between alpha and gamma power, which is flipped for convenience in Fig 9 but indicated in red. Interestingly, change in delta power does not correlate significantly with changes in any of the other 8 measures, confirming again that changes in complexity do not simply reflect changes to the overall power spectrum.

### Grouping EEG into lobes

To assess whether changes in complexity during LOC occurred across the whole cortex, or whether more local changes could be identified, we analysed the measures on each of the four lobes separately. The electrodes were divided into four groups, each corresponding to one of the four main anatomical lobes: temporal, occipital, frontal and parietal, see Fig 2. Within each lobe, 25 channels were automatically chosen via k-medoids and from them the measures LZc, SCE and ACE computed in the same way as for the whole cortex.

**Fig 10:**
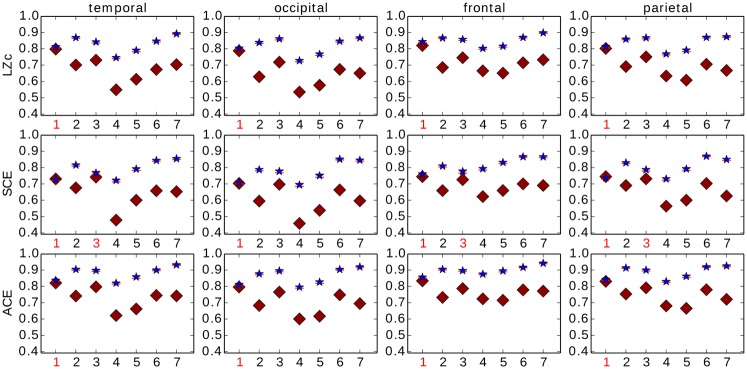
Analysis of 25 k-medoids chosen electrodes for each of four lobes. Blue star is WR, red diamond is LOC. The red error bars indicate standard error. LZc and ACE score for all subjects higher for WR as opposed to LOC, SCE does so for 6/7 subjects. The measures behave similarly for different brain regions. If the effect size Cohen’s *d* < 0.8 for the scores of a given subject, the subject’s label is printed in red.

LZc and ACE scored consistently higher for WR than for LOC for all four lobes for all subjects. This was with Cohen’s *d* > 0.8 for all lobes for all subjects, except subject 1, see Fig 10. SCE scored higher for WR than for LOC for 6 out of the 7 subjects for each lobe, with Cohen’s *d* > 0.8 for all subjects and lobes, except subject 1 for all lobes and except subject 3 for all lobes other than the occipital lobe (see red subject labels in Fig 10). Thus the discriminative power of the measures was similar when computed across a single lobe to when computed across the whole cortex.

### Frequency filtered whole cortex recordings

We also analysed the LZc, SCE and ACE measures on the whole cortex data restricted to different frequency bands, using the same bands as before and also a high-pass filter, excluding all frequencies below 1*Hz*. Butterworth filters were applied to restrict the signal to the respective frequency bands prior to computing the complexity measures. The results for the 25 electrodes—automatically chosen via k-medoids across the whole cortex—are summarized in Table 2.

**Table 2:** Effect size comparison per measure and frequency band for WR/LOC.

	delta	theta	alpha	beta	gamma	> 1*Hz*
LZc	5 2 0	1 4 2	5 2 0	5 2 0	6 1 0	5 2 0
SCE	6 1 0	3 3 1	4 2 1	**7 0 0**	6 0 1	6 1 0
ACE	**7 0 0**	1 4 2	4 3 0	6 1 0	**7 0 0**	5 2 0

For each measure and frequency-band-filtered input, the three numbers display how many subjects out of 7 had higher score for WR than LOC with Cohen’s *d* > 0.8 (left digit), no substantial difference, *d* < 0.8, between WR and LOC (middle), and lower values in WR when compared to LOC with Cohen’s *d* > 0.8 (right). High-pass-filtered input data are labelled by > 1*Hz*.

The findings suggest that the elevated complexity in the WR state arises more from activity in low and high frequency bands (delta, beta, gamma) than from intermediate frequency bands (theta, alpha), and that complexity is not just a property of the low frequency components of the spectrum.

## Discussion

We have analysed three different complexity measures on spontaneous EEG data from subjects undergoing propofol-induced general anaesthesia: Lempel-Ziv complexity (LZc), synchrony coalition entropy (SCE) and amplitude coalition entropy (ACE). All three of these measures robustly distinguished loss-of-consciousness (LOC) from wakeful resting (WR) on the broadband signal, giving higher mean values for WR as compared to LOC across subjects, a range of segment lengths, and number and location of electrodes (individual lobes versus whole cortex). On analyses restricted to specific frequency bands, the discriminative power of the complexity measures was highest for low and high frequency bands (delta, beta, gamma), and lower for intermediate bands (theta, alpha). The measures ACE and LZc also had some ability to discriminate mild sedation (MS) from WR.

We combined several approaches to verify that LZc, SCE and ACE capture more than just spectral changes between the states. First, we found that re-normalizing the measures by values obtained after phase randomization did not affect the results (see section ‘Control for changes in power spectrum’ in S1 Document). In addition, when comparing the discriminative power (as measured by AROC) of the three complexity measures with that of normalized spectral power (on the non-randomized data) we found that normalized delta power discriminated between the states WR and LOC as well as the three complexity measures did, but it could not discriminate between MS and WR (see Figs 8, 7 and S3 Fig in S1 Document and Table 1), unlike LZc. Further, all three complexity measures tend to behave monotonically with respect to depth of sedation (see Fig 7), unlike delta or gamma power (see S3 Fig in S1 Document). Normalized spectral power in other bands all had weaker discriminative power for WR/LOC. Finally, we did not see significant correlations between the change in delta power and changes to any of the complexity measures, amongst the 7 subjects (Fig 9).

The consistency of our findings across the four cortical lobes, over different numbers of channels, segment lengths, or normalization (spectral-profile preserving or not) shows that the decrease in spontaneous EEG complexity during general anaesthesia is very robustly measurable. In ongoing work we are exploring the behaviour of these measures on a finer spatial scale to see at what level, if any, regional differences can be detected.

What exactly have we measured? All the complexity measures we have considered are computed from binary matrices of activations (LZc, ACE) or synchronies (SCE). Each of the measures quantifies the level of randomness in such a matrix. We verified explicitly that if the matrix is a random matrix with some time-series identical, then all the measures decrease monotonically with the number of identical time-series (see Fig 5). LZc counts the number of distinct patterns in the concatenated observations, or equivalently computes how incompressible the matrix is, while ACE and SCE are based on the average surprise (entropy) of a single *n* × 1 observation drawn at random from the matrix (*n* being the number of time series). Thus, the measures quantify the degree of randomness of a matrix in slightly different ways, diverging slightly for intermediate levels of randomness, but under normalization, all tend to zero in the limit of an infinite matrix with every time-series the same, and all score one in the limit of an infinite matrix in which each entry is independent and has equal chance of being one and zero.

The entropic brain hypothesis of Carhart-Harris et al [51] states that there is correlation between the degree of overall randomness (entropy, differentiation) in brain dynamics and ‘vividness of cognition’, locating brain states such as coma, anaesthesia and deep sleep at relatively low entropy, wakeful rest at intermediate entropy and REM sleep and psychedelic states at relatively high entropy [51]. In support of this, simple randomness measures applicable to a single time series, such as approximate entropy [52] and permutation entropy [31], have been found to decrease during anaesthesia and sleep [27, 28, 32, 34]. Our results are consistent with these findings as well as with theories of consciousness based more explicitly on some conceptualisation of ‘complexity’ [13]. It is clear that our three measures are capturing differentiation in the sense of signal diversity: if all regions were to behave identically, then amplitude and synchrony coalitions would not vary over time (ACE and SCE would score low), and there would be few distinct patterns in the binary matrices (LZc would score low).

To what extent can we say that the measures reflect *complexity*, in the sense of co-existing differentiation and integration amongst the EEG signals [3]? Regarding integration, variations over time to the set of active or synchronous channels could arise in the absence of causal interactions; for instance, each channel could be exhibiting its own individual chaotic dynamics, and be evolving in isolation. Thus the measures LZc, SCE and ACE would clearly not in general for any system capture integration. However, structural constraints mitigate against fully random activity in the context of the thalamocortical system. Given that most cortical regions tend in general to receive strong driving input from sub-cortical regions [53], it would seem that diverse activity across EEG channels is likely to require a certain amount of functional integration [18, 21, 36]. Thus LZc, SCE and ACE, when applied to multi-dimensional EEG recordings of brain activity, may cautiously be considered to also correlate with integration in EEG dynamics, and therefore to track complexity in the sense of simultaneous differentiation and integration.

Our simulation with the Kuramoto model demonstrated that SCE, with its analysis of phase (as opposed to amplitude) synchrony, can exhibit different behaviour from LZc and ACE (see Fig 6). We varied a parameter that controls the overall level of synchrony, and found that SCE peaked strongly at an intermediate level of overall synchrony (as one would expect), while LZc and ACE actually exhibited a small dip where SCE peaked. This simulation, together with the slightly different results for the three measures when applied to EEG (see Fig 7), demonstrate that at least two distinct ‘flavours’ of complexity were decreasing in the spontaneous EEG during anaesthesia. In further work we will conduct additional simulations to examine more closely situations in which these three measures converge and diverge.

Our results complement those of Casali et al [21] who measured Lempel-Ziv complexity of the EEG response to transcranial magnetic stimulation (TMS). In their paradigm, high levels of Lempel-Ziv complexity corresponded to conjoined differentiation and integration by virtue that a high score could only be obtained when the neuronal activity in response to the magnetic perturbation spreads far (integration) and evolves in a non-stereotypical way (differentiation). Their variant measure, called the Perturbational Complexity Index (PCI), also scored consistently higher for WR than for propofol anaesthetized subjects (LOC) [21], in addition to also scoring relatively low values for other unconscious states, namely, non-rapid eye movement sleep and vegetative state.

In summary, we have demonstrated a correlation between level of sedation and three distinct complexity measures during propofol-induced general anaesthesia. Testing the measures on data from other manipulations of conscious level will build up a more complete indication of the specificity and sensitivity of these measures to the level of consciousness associated with diverse states, and also whether they can act synergistically with other indices [36]. Since we did not find any complexity measure to correlate significantly with delta power, we expect the complexity measures to combine synergistically with a coarse analysis of the power spectrum. This would be in line with recent work by Sitt et al. [36] analysing auditory evoked potentials in EEG from traumatic brain injury patients. They combined spectral measures with measures of the complexity of a single time-series to achieve greater discriminative power (as measured by area under the ROC curve in classifying the states of traumatic brain injury patients).

Other theory-based indices that could be explored in conjunction with the complexity measures described here include ‘causal density’ [13] and various versions of integrated information (as measured by the quantity ‘phi’ [14]), integration measures based on long-range functional connectivity such as weighted symbolic mutual information [54], integration measures based on microstate properties across channels of spontaneous EEG activity, as recently applied to classify disorders of consciousness patients by Fingelkurts et al [7], and global graph-theoretic measures such as ‘efficiency’ of connectivity graphs [55]. Moreover, the computation of the LZc and ACE measures based on a simplification of the continuous EEG to a binary signal necessarily discards some information content from the signals analyzed. It is possible that utilising a transformation of the continuous signal into a repertoire of ‘symbols’, as in [54], could increase discriminative power.

There are now a number of candidate ‘neural correlates of consciousness’ [56]. We have added to the list multi-dimensional complexity of spontaneous EEG. Our measures derive from theory and thus are ‘explanatory correlates’ [57], are quick and easy to compute, and do not involve the need to stimulate or perturb the brain.

## Supporting Information

S1 Document Supplementary material. This document contains a more detailed discussion of the Lempel-Ziv compression variants as well as results for control analysis as desribed in the text.
(PDF)

S1 Code Python Script. With this python script the complexity measures LZc, SCE and ACE can be computed from continuous multidimensional time series.
(ZIP)
