# Supplementary material for: Complexity of Multi-Dimensional Spontaneous EEG Decreases during Propofol Induced General Anaesthesia
Source: PLoS One. 2015 Aug 7;10(8):e0133532. doi: 10.1371/journal.pone.0133532 (PMC4529106; doi:10.1371/journal.pone.0133532)
Supplement: S1 Document — This document contains a more detailed discussion of the Lempel-Ziv compression variants as well as results for control analysis as desribed in the text. (PDF) [file pone.0133532.s001.pdf]

Supplementary material for
Complexity of multi-dimensional spontaneous EEG decreases during propofol induced general anaesthesia

Michael M. Schartner*, Anil K. Seth, Quentin Noirhomme, Melanie Boly,
Marie-Aurelie Bruno, Steven Laureys, Adam B. Barrett
*m.schartner@sussex.ac.uk (correspondence)

CONTENTS

I LZ measures 1
I-A LZ Compression and data concatenation 1
I-B Differences between LZc and PCI 1
II Dependence on number of electrodes 2
III Dependence on segment length 2
IV Control for changes in power spectrum 2
V Results for alternative measures 2
References 4

I. LZ MEASURES

A. LZ Compression and data concatenation

There are several different versions of the Lempel-Ziv-Welch complexity c of a sequence. The alogrithm we used for computing the measure LZc is the following. Let S be a binary sequence $S = s_1 s_2 \dots s_n$. Then c is the number of distinct 'words' in the sequence, obtained as follows. The dictionary is taken to contain the words '0' and '1' already at the outset. The next word to go in the dictionary is the first two digits of S. Then the algorithm proceeds iteratively: suppose we have already obtained the first r words $w_1, \dots, w_r$ from $s_1 s_2 \dots s_k$. Then w_{r+1} is the shortest sequence $\sigma_k^p =: s_k s_{k+1} \dots s_{k+p-1}$ such that $\sigma_k^p \notin \{w_1, \dots, w_r\}$; that is, the next word is the shortest sub-sequence of S that starts from the final digit of the previous word and is not already in the dictionary. Other versions of the algorithm, such as the one used by Casali et al [3], obtain the next word by starting from the first digit after the end of the previous word, but we found the version described here quickest and easiest to implement, and found that choice of algorithm had no effect on the results. An example sequence is $S =$ 01011010001101110010, which gives the list of words $\{0, 1, 01, 10, 011, 101, 100, 00, 0110, 0111, 1001\}$.

For a string consisting only of zeros, a relatively small dictionary is obtained: if $S =$ 00000000000000000000 then the dictionary is $\{0, 1, 00, 000, 0000, 00000, 000000\}$. We implemented the algorithm in Python (adapted from [1]), see Supplementary Material (Code S2) for the code.

As we analysed multidimensional time series, there are several ways to transform the binarized data matrix into one sequence. The following variants were tested. Firstly, concatenating the data time series by time series. Secondly, concatenating the data observation by observation (to obtain the measure LZc; this was also Casali et al's choice [3]) and thirdly, computing the compressibility for each time series separately and then averaging the results across all series (to obtain the measure we call LZsum; this gives Sitt et al's, K complexity [4], yet for binary input). The way in which the data were concatenated into one sequence (time series by time series, observation by observation, or no concatenation - as for LZsum) didn't make much difference to the results yet MS/WR was more clearly discriminated by LZc as opposed to LZsum (compare Figure S3 with Figure 7 in main text).

B. Differences between LZc and PCI

The LZc measure used here differs from Casali et al's [3] PCI in several ways. Source modelling of the EEG was applied by Casali et al [3] before computing PCI, whereas we computed LZc on Laplace-filtered scalp signals. PCI captures compressibility of the EEG response to TMS whereas our LZc is applied to spontaneous EEG. Reflecting this, there is a difference in obtaining the binarization threshold. We used for LZc the mean of the absolute value of the analytic signal whereas Casali et al used the 99th percentile of this during the prestimulus activity (supp. mat. of [3]) so that a 1 corresponded to significant perturbation. PCI is computed across a $0.3sec$ data segment that starts directly after magnetic stimulation; our results were most robust for segment lengths of between $0.2sec$ and $20sec$, although segment lengths shorter than $1sec$ substantially affected the results for one subject only (see section 'Dependence on segment

length'). For PCI, the time-series are ordered with the most often significantly activated sources at one end and the least often significantly activated sources at the other end. For both LZc and PCI, the binarised data are concatenated in the same way into a sequence. For LZc a dictionary of binary words is obtained from the sequence by applying the Lempel-Ziv-Welch algorithm [1], whereas Casali et al use the algorithm given in [5]. This dictionary size - reflecting the compressibility of the binary sequence - is normalized in the case of LZc by the dictionary size for the same input shuffled in time (as explained in main text, Methods Section 'Lempel-Ziv complexity'). Casali et al [3] normalize by the asymptotic upper bound: $LH(L)/log_2(L)$, where L is the total number of binary matrix entries and $H(L)$ the source entropy of this matrix (supp. mat. [3]).

II. Dependence on number of electrodes

In order to test whether the number of channels of the input data affected the behavior of the measures for the state pair WR/LOC, we tried 5, 10, 50, and 100 k-medoids selected electrodes in addition to the 25 (see Figure S1). We found all three measures' scores to be higher for WR than LOC for all tested channel numbers. For all subjects and measures, the differences between WR and LOC had high effect sizes (Cohen's $d > 0.8$) except for one subject for all three measures and channel numbers and two more subjects for measure SCE and certain channels (see subject label color in Figure S1). Despite varying effect sizes, this control suggests that the measures' scores are across subjects higher for WR than LOC for a broad range of channel numbers.

III. Dependence on segment length

As a second control we tested whether segment length influences the behavior of our measures. Analysed across segments of length other than $10sec$, we found for ACE, SCE and LZc very similar results in almost all cases for all tested segment lengths (0.2, 0.4, 0.8, 1.2, 2, 4, 6, 10 and 20 seconds); except for one subject the behavior of the measures did not vary with segment length (Figure S2). That is, the measures scored higher for WR than LOC with high effect size (Cohen's $d > 0.8$) across all tried segment lengths for 6 of the 7 subjects. Subject 1 was an exception, with LZc, SCE and ACE showing the same behavior as for the other subjects only for segment lengths greater than 4 seconds. In sum, this control shows that the behavior of the measures is robust across a large range of segment lengths.

IV. Control for changes in power spectrum

To test directly whether changes in the complexity measures arose from more than just changes in the spectral profile of the input signal, we compared two different types of normalisation of the measures. First, as used for the results in the main text, 'time shuffling', obtained from surrogate data for which the measure reaches its upper bound, but that has a flattened spectral profile (see Sections 'Lempel-Ziv complexity' and 'Coalition entropy measures'). Second, 'phase shuffling', obtained from surrogate data with a conserved spectral profile. We find qualitatively similar results for both types of normalisation, and thus confirm that changes in the complexity measures reflect more than just spectral changes.

The 'phase shuffling' normalisation is obtained from phase-randomized surrogate data as follows. From the complete data from a given subject in a given state, a segment is randomly chosen. Each time series of that segment is expressed as a superposition of sinusoids using fast-Fourier transform. Then the phase of each sinusoid is independently randomly changed, before applying inverse Fourier transform. The complexity measure is computed for 100 such phase-randomized data segments. The mean of these 100 scores is then used to normalize the measure's score for the original data segments. Averaging over 100 such surrogate data segments suffices to obtain negligible randomness in the normalisation factor.

Figure S4 is a comparison of the three complexity measures' scores per subject for the two normalisation methods. As can be seen when comparing the measures' results subject by subject, the qualitative behaviour of all three measures is very similar under both normalizations, implying that the signal complexity we measure is not trivially connected to spectral properties.

V. Results for alternative measures

Besides LZc, SCE and ACE, the following alternative measures scored for certain state pairs consistently different across subjects (compare with Table 1 and Figure 8 of main text). Figure S3 displays that normalized delta power and sumCov (average correlation of the signals) are both consistently higher for LOC than for WR across subjects. Note that neither of them scores for MS consistently in between WR and LOC, whereas LZc, SCE and ACE do so for all except subject 2 for SCE. The Lempel-Ziv variant LZsum and gamma power also discriminate WR/LOC well, except for the first subject, see Figure S3. Interestingly, LZsum performs less well in discriminating MS/WR, showing that the way in which the binary matrix was concatenated influenced the outcome. I.e. the observation-by-observation concatenation used for LZc results in capturing randomness across channels as opposed to capturing average randomness per channel, as used in the computation of LZsum.

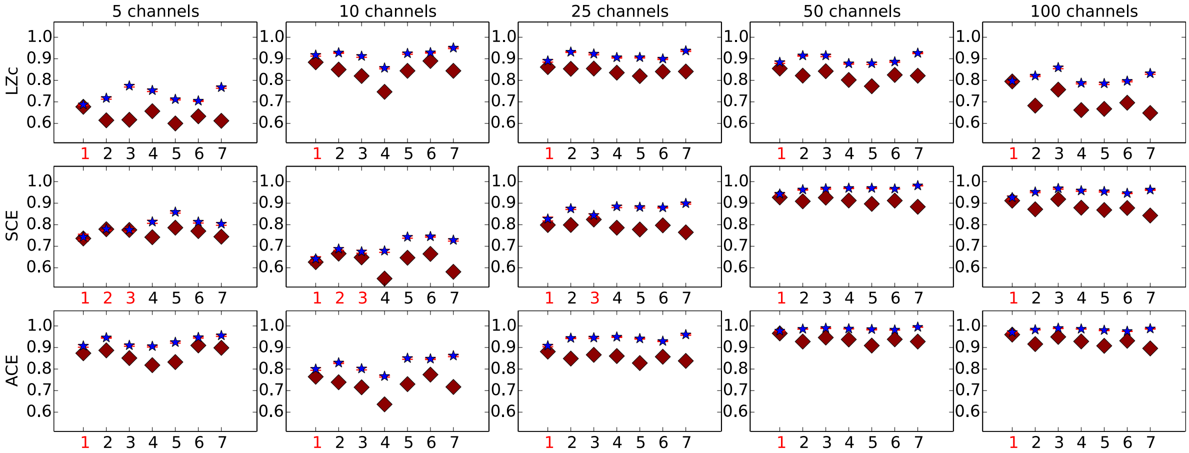

Fig. S1: **WR (asterisks) and LOC (diamonds) scores of LZc, SCE and ACE for different numbers of electrodes.** $\{5, 10, 25, 50, 100\}$ electrodes were chosen via k-medoids from the whole cortex. For all subjects and channel numbers, the score of any of the three measures is higher for WR than for LOC. Red subject labels indicate small effect size, i.e. Cohen's $d < 0.8$. For LZc and ACE only subject 1 shows small effect size for any channel number. For SCE up to three subjects show small effect size for certain channels.

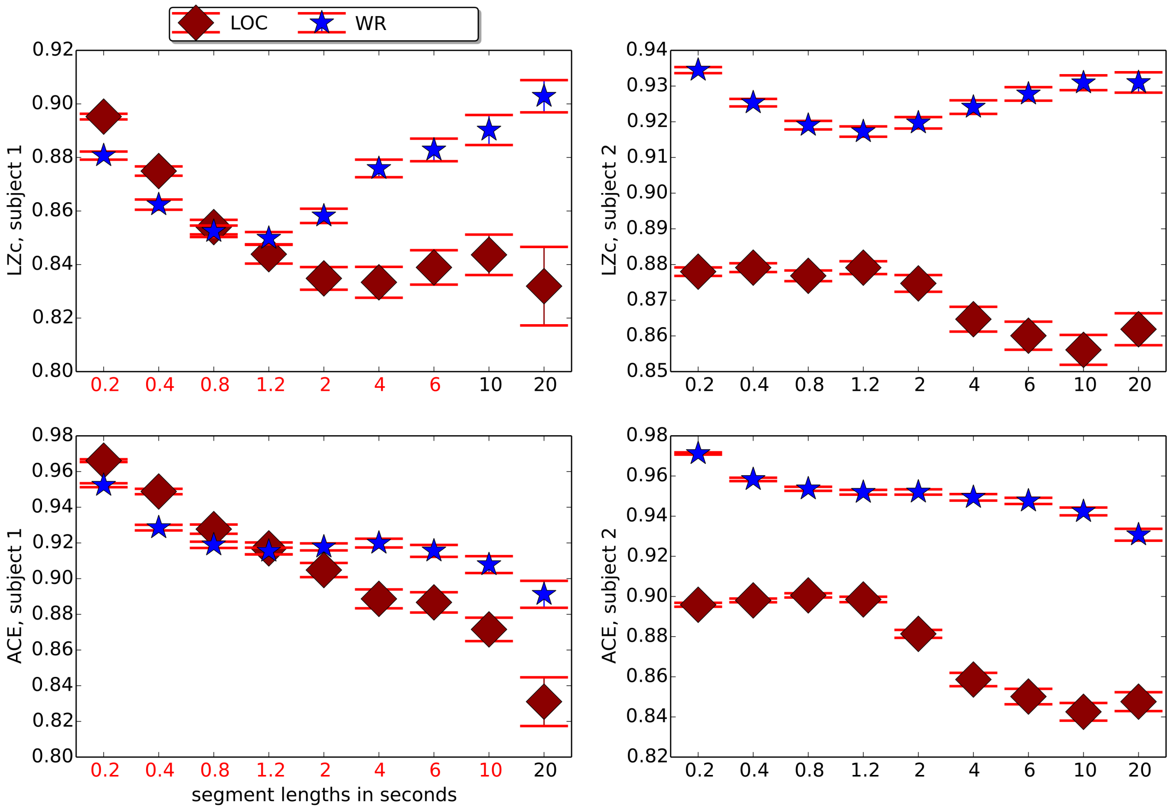

Fig. S2: **Sensitivity of the measures to segment length.** Segment lengths are indicated in seconds, colored red if the difference between WR and LOC has effect size $d < 0.8$. There is one out of 7 subjects, subject 1, for which the segment length changed the measures LZc, SCE and ACE from being greater for WR than for LOC to the opposite, i.e. smaller for WR than for LOC. This change happened at a segment length close to 1 second. This can be seen in the left two panels, which indicate respectively the score of LZc and ACE, each for the two states WR and LOC for subject 1. For the remaining 6 of the 7 subjects, the segment length of the input had no influence on the order of the LZc (SCE, ACE) scores for WR and LOC, as is exemplified in the right two panels with subject 2. (Similar results hold for SCE, not shown).

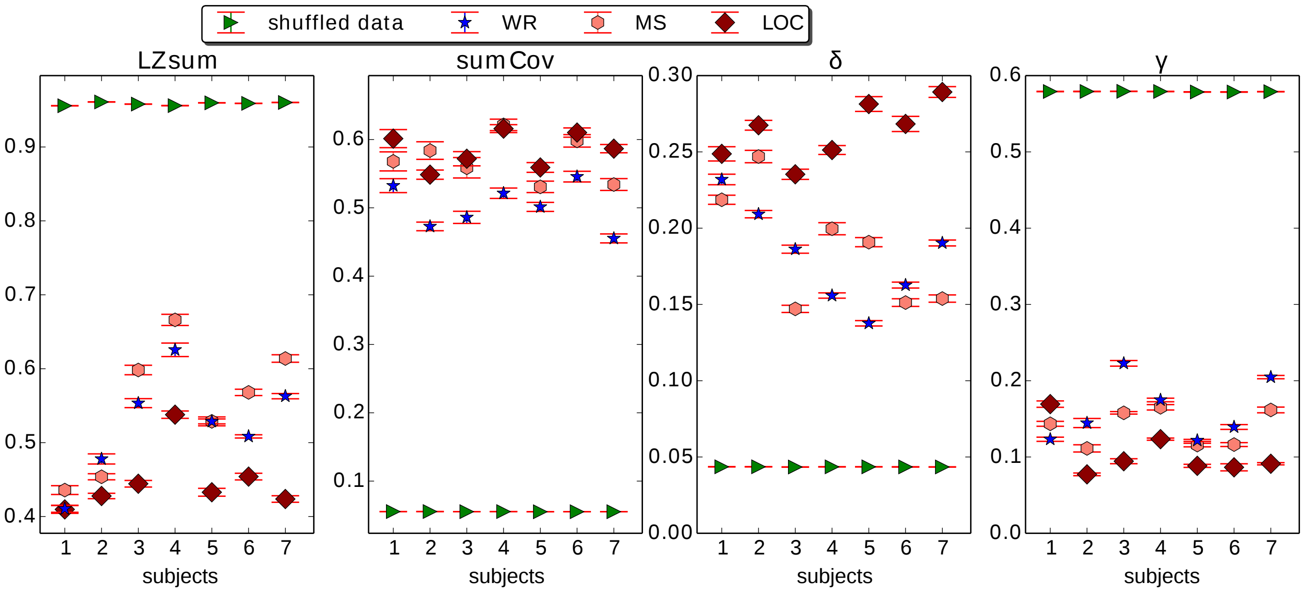

Fig. S3: **Mean LZsum, sumCov, normalized delta power and normalized gamma power**, computed for broadband signal of 25 channels from the whole cortex, 10sec segments, i.e. the same input as for Table 1 and Figure 8 of main text. States shown are WR, MS, LOC and shuffled data. sumCov and delta power discriminate WR/LOC consistently across subjects, whereas neither scores for MS in between WR and LOC consistently across subjects. LZsum separates WR/LOC for 6/7 subjects and has poor discrimination for MS/LOC, unlike LZc. Error bars indicate standard error across segments.

References

[1] http://rosettacode.org/wiki/LZW_compression#Python

[2] Perez F, Granger B (2007) IPython: A System for Interactive Scientific Computing. Computing in Science and Engineering, vol. 9, no. 3, pp. 21-29, http://ipython.org

[3] Casali A, Gosseries O, Rosanova M, et al. (2013) A Theoretically Based Index of Consciousness Independent of Sensory Processing and Behavior. Sci. Transl. Med. 5, 198ra105.

[4] Sitt J, King J, et al. (2014) Large scale screening of neural signatures of consciousness in patients in a vegetative or minimally conscious state. Brain 2014;137(pt 8):2258–2270.

[5] Kaspar F, Schuster H (1987) Easily calculable measure for the complexity of spatiotemporal patterns. Phys. Rev. A 36, 842–848.

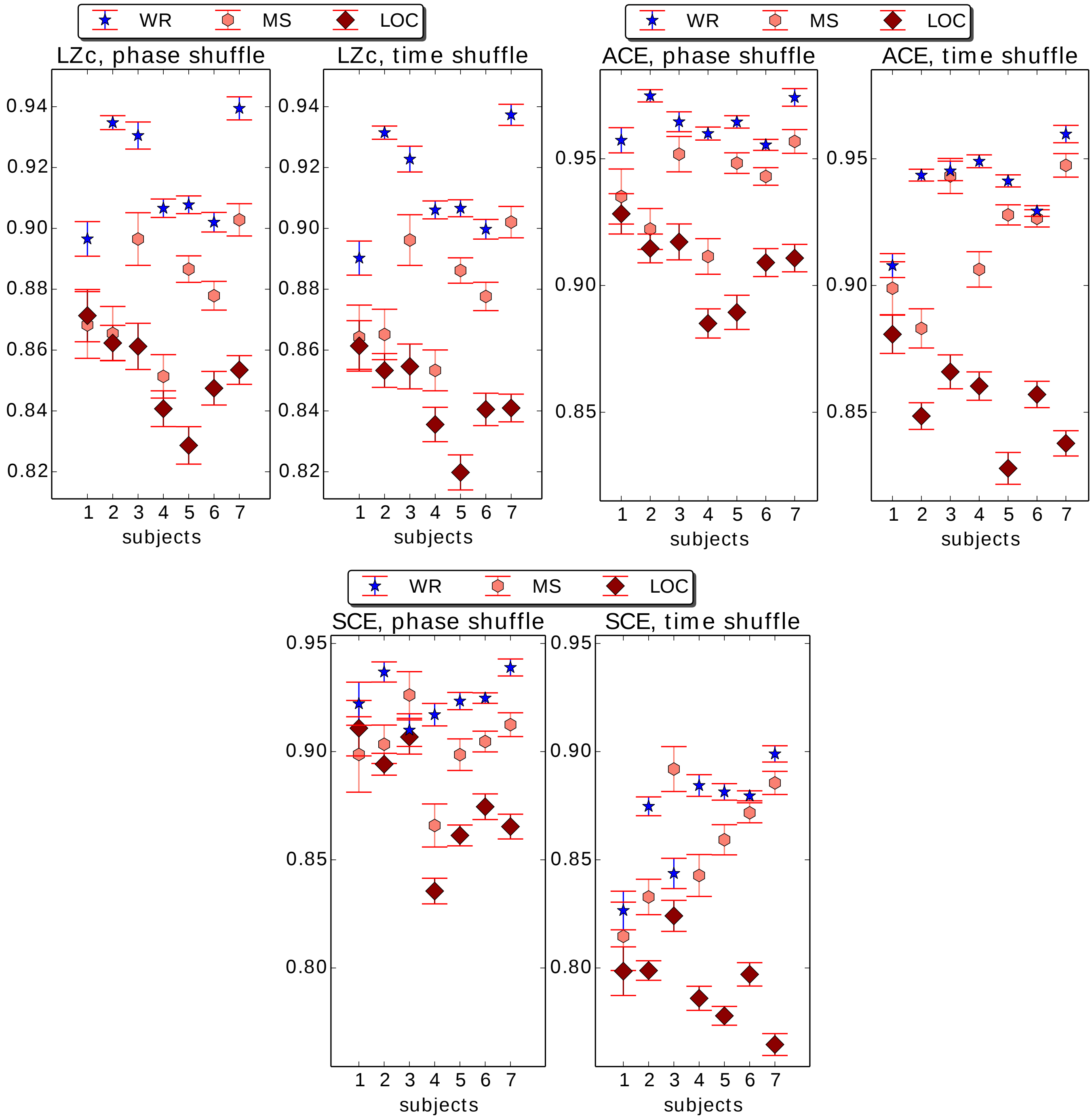

Fig. S4: **Invariance of the measures' qualitative behaviour under spectral-profile-preserving normalisation.** LZc, SCE and ACE computed as averages over multiple 10sec segments of EEG of the 7 subjects before and during anaesthesia, as described in caption for Figure 7 in main text, yet here for two different normalisations. The titles indicate which measure and which normalisation is displayed (see main text for details). The measures' qualitative behaviour is very similar for the two normalisations.
